# Guided growth of the trochanteric apophysis combined with soft tissue release for Legg–Calve–Perthes disease

**DOI:** 10.1007/s11751-014-0186-y

**Published:** 2014-02-23

**Authors:** Peter M. Stevens, Lucas A. Anderson, Jeremy M. Gililland, Eduardo Novais

**Affiliations:** 1 Department of Orthopaedics, University of Utah, Salt Lake City, UT USA; 2 Department of Orthopaedics, University of Colorado, Boulder, CO USA; 3 School of Medicine, Primary Children’s Hospital, University of Utah, 100 Mario Capecchi Drive Suite 4550, Salt Lake City, UT 84113 USA

**Keywords:** Legg–Calve–Perthes disease, Trochanteric arrest, Guided growth, Coxa brevis, Containment

## Abstract

During the initial fragmentation stage of Perthes disease, the principle focus is to achieve containment of the femoral head within the acetabulum. Whether by bracing, abduction casts, femoral and/or pelvic osteotomy, the goals are to maximize the range of hip motion and to avoid incongruity, hoping to avert subsequent femoro-acetabular impingement or hinge abduction. A more subtle and insidious manifestation of the disease relates to growth disturbance involving the femoral neck. We have chosen to tether the greater trochanteric physis, combined with a medial soft tissue release, as part of our non-osteotomy management strategy for select children with progressive symptomatology and related radiographic changes. In addition to providing containment, we feel that this strategy addresses potential long-range issues pertaining to limb length and abductor mechanics, while avoiding iatrogenic varus deformity caused by osteotomy. This is a retrospective review of 12 patients (nine boys, three girls), average age 7.3 years old (range 5.3–9.7), who underwent non-osteotomy surgery for Perthes disease. An eight-plate was applied to the greater trochanteric apophysis at the time of arthrogram, open adductor and iliopsoas tenotomy, and Petrie cast application. We compared clinical and radiographic findings at the outset to those at an average follow-up of 49 months (range 14–78 months). Six plates were subsequently removed; the others remain in situ. Eleven of twelve patients experienced improvement in pain, and alleviation of limp and Trendelenburg sign at latest follow-up. The majority had improved or maintained range of motion and prevention of trochanteric impingement demonstrated by near normalization of abduction. Neck-shaft angles, Shenton’s line, extrusion index, center edge angles and trochanteric height did not change significantly. One patient underwent subsequent trochanteric distalization and no other patients have undergone subsequent femoral or periacetabular osteotomies. Leg length discrepancy worsened in four patients and was treated with contralateral eight-plate distal femoral epiphysiodesis. As a group the mean leg length discrepancy did not change significantly. There were no perioperative complications. six trochanteric plates were subsequently removed after an average of 43.7 months (range 28–69) due to irritation of hardware; the others remain in situ, pending further growth. We employed open adductor and iliopsoas tenotomy and Petrie cast application and guided growth of the greater trochanter as a means of redirecting the growth of the common proximal femoral chondroepiphysis. The accrued benefits of preventing relative trochanteric overgrowth with a flexible tether are the avoidance of iatrogenic varus and weakening of the hip abductors. The goals are to preserve abductor strength and avoid trochanteric transfer or intertrochanteric osteotomy.

## Introduction

Containment of the femoral head within the acetabulum is a common treatment strategy in Legg–Calve–Perthes disease (LCPD). Through alleviation of contractures and restoration of motion, the ultimate goals are to ensure a relatively spherical femoral head and improved congruency of the hip. A number of treatment modalities for surgical containment include bracing [1], proximal femoral varus osteotomy (PFVO) [2], innominate [3] and shelf osteotomy [4]. However, there is no consensus regarding which surgical procedure is most efficacious [4, 5]. In addition, neither PFVO nor a pelvic osteotomy addresses the relative growth disturbance of the proximal femoral and trochanteric growth plates usually present in LCPD [6]. In fact, the downstream effect of varus at the hip via innominate or femoral osteotomy may exacerbate femoro-acetabular impingement.

**Fig. 1:**
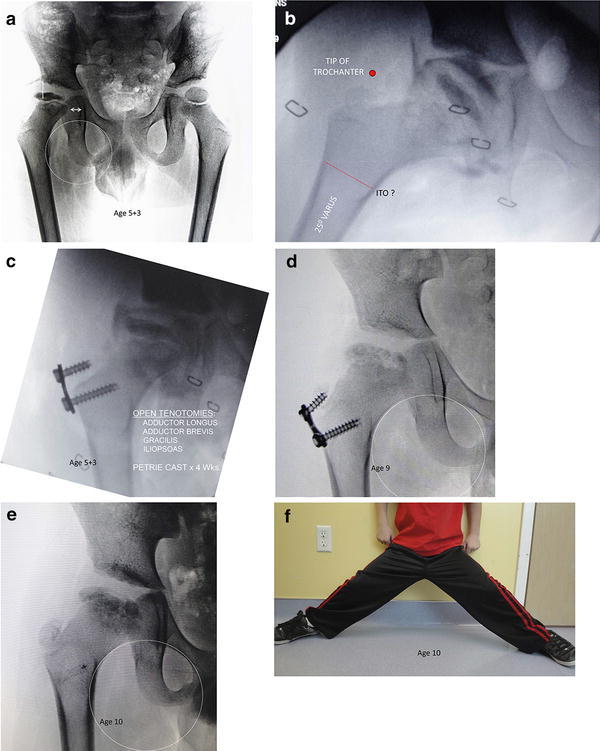
**a** This patient developed subluxation and “head at risk” signs, during a 6 month period of observation. Note the break in Shenton’s line, medial clear space widening, and lateral uncovering of the femoral head. Evolving acetabular dysplasia is also evident. **b** An arthrogram in 25° of abduction demonstrates good containment, but unacceptable elevation of the greater trochanter. Furthermore, intertrochanteric osteotomy would shorten the limb and sacrifice abduction accordingly. **c** In lieu of an osteotomy. For containment, we chose to perform open adductor tenotomies, including the iliiopsoas, tethered the greater trochanter, and placed him in a Petrie cast for 4 weeks. **d** By age 9, note the divergence of the screws, indicating differential medial growth by holding the greater trochanter in abeyance. He had full abductor strength and restoration of Shenton’s line. The plate was removed. **e** Presumably as a result of strong abductors, the acetabular dysplasia has resolved. **f** Age 10: he is asymptomatic, with full range of motion and equal limb lengths. He will be seen on an annual basis

The medial two-thirds of the proximal femoral chondroepiphysis has a common intracapsular blood supply. Consequently, ischemia may result in an aspherical femoral head and a short, broad femoral neck. The greater trochanter, which has a separate extracapsular blood supply, becomes relatively “overgrown” and prominent, reducing the effective abductor lever arm. Concurrently, with *coxa magna*, the center of rotation of the femoral head is effectively displaced distally, further exacerbating the problem (Fig. 1). The resultant deformity—*coxa brevis*—contributes to abductor weakness, Trendelenburg gait and secondary acetabular dysplasia while producing limb length inequality and a cam-type femoro-acetabular impingement. Therefore, avoiding relative overgrowth of the greater trochanter is an important goal to include in the treatment for LCPD.

Epiphysiodesis of the greater trochanter by drilling and curettage potentially arrests half of the growth of the greater trochanter [7]. The most widely used surgical technique involves a modification of the Phemister method [7] combined with a PFVO [8]. The role of trochanteric growth arrest in the management of patients with LCPD, however, remains controversial [6, 9].

Recognizing the insidious and detrimental effects of progressive, relative overgrowth of the greater trochanter often exacerbated by osteotomy, we have adopted a new approach for containment. The goals of this study were to (1) describe our non-osteotomy treatment protocol that involves arthrogram of the hip, adductors and iliopsoas tenotomy, epiphysiodesis of the greater trochanter with a tension plate and application of a Petrie cast; and (2) report the preliminary clinical and radiographic outcomes after this combined procedure.

## Methods

We conducted an IRB approved, retrospective review of the medical records and radiographs of 12 patients (9 boys, 3 girls) with LCPD. The age at diagnosis of LCPD ranged from 4 to 9 years (mean 6 years). The mean chronologic age at index surgery was 7.3 years (range 5.3–9.7 years) (Table 1). Each patient had experienced loss of motion and increasing pain and limping, despite initial conservative management that included activity restriction and non-steroidal anti-inflammatory drugs. Serial radiographs demonstrated whole femoral head involvement in the fragmentation stage, often with a break in Shenton’s line.

**Table 1:** Demographic data (*n* = 12 hips in 12 patients)

	Finding
Age at time of LCPD diagnosis (years)^a^	6 (1.6)
Age at time of operation (years)^a^	7.3 (1.5)
Gender (M/F)	9/3
Height (inches)^a^	46 (5)
Weight (lbs)^a^	52 (11)
Affected hip (R/L)	4/8

^a^Data presented as mean with SD in parenthesis

Early in the series, two of the children had isolated guided growth of the greater trochanter, without tenotomy or casts. Our current operative protocol includes the following: (1) hip arthrogram, (2) open proximal tenotomy of the adductors longus and brevis, the gracilis and iliopsoas, (3) guided growth of the greater trochanter by insertion of a tension band two-hole plate (Fig. 1c), and (4) application of a Petrie cast for 4 weeks. The rationale for including the iliopsoas is to gain additional inward rotation and abduction.

The surgery was accomplished under a general anesthetic; the post-operative pain regimen included epidural catheter or patient-controlled analgesia for 24 h. Patients were then weaned to oral medication and discharged, encouraging weight-bearing, as tolerated, with crutches. The cast was removed 4 weeks following surgery. Physical therapy was then prescribed for strengthening and gait training.

Clinical outcome was assessed before and at regular intervals after surgery and included evaluation of gait, bilateral hip range of motion, limb lengths and the Trendelenburg test [10]. The impingement testing was performed with 90° of hip flexion, adduction and internal rotation and was positive if it caused discomfort [11]. In addition to the standard supine examination for range of motion, the degree of abduction was measured with the patient observed standing and attempting to do “the splits” (Fig. 1f).

Radiographic outcomes were determined by preoperative, post-operative as well as biannual radiographs after surgery; these included a standing anteroposterior view of the pelvis. Acetabular coverage was evaluated by measuring extrusion index (EI: head width divided by width of head covered by the acetabulum) [12] and lateral center edge angles (LCEA) [13]. To assess the neck-shaft angle and see the greater trochanter better, an anteroposterior radiograph of the involved hip was obtained with the limb rotated inward 15°. We measured neck-shaft angles and noted the degree of femoral head involvement, stage of the disease (fragmentation versus healing) and relative limb lengths, and evaluated Shenton’s line [14]. We determined the center-head trochanteric distance (CTD), the height of the tip of the greater trochanter relative to the center of the hip, and deemed a positive value as the center of the head being above the tip of the greater trochanter [15] (Fig. 1d).

Data were analyzed by an independent statistician using commercially available software (STATA version 11, College Station, TX). Student’s *t* test was used for comparing all continuous variables. The chi-squared test was used to compare the binary variables if the expected frequencies were all greater than five. Fisher’s exact test was used to compare those binary variables where the expected frequencies were not adequate for the chi-squared test.

## Results

Patients had an average follow-up of 49 months (range 14–78 months). Preoperatively, all patients had a limp and positive Trendelenburg sign and limited abduction. They were followed at 3- to 6-month intervals for clinical and radiographic evaluation and up to the time of most recent examination. At the time of most recent examination, all patients had improvement in their pain and were without activity restriction. One patient had a slight limp and Trendelenburg sign at latest follow-up which was still a significant improvement compared to a majority of patients having these findings preoperatively. In the standing position, there was significant improvement in symmetric abduction at latest follow-up (mean 58° vs. 30°; *p* < 0.005). Additionally, internal rotation was significantly improved at latest follow-up (mean 43° vs. 21°; *p* = 0.012), while external rotation did not change significantly. Significant improvement was also seen in hip impingement as nine patients had findings of impingement preoperatively compared to only two patients at latest follow-up (*p* = 0.012, Table 2).

**Table 2:** Clinical and radiographic measures (*n* = 12 hips with mean follow-up of 49 months)

	Pre-operative	Post-operative	*p* value
Radiographic measures			
Neck-shaft angle (°)	126 (121–130)	123 (119–128)	0.052
Center-trochanter distance (mm) (+ = superior, − = inferior)	5 (−3 to 10)	3 (−2 to 9)	0.510
Lateral center edge angle (°)	13 (8–18)	12 (7–16)	0.406
Extrusion index (%)	75 (72–79)	70 (63–76)	0.127
Disruption of Shenton’s Line	5	1	0.155
Leg length discrepancy (mm)	12 (7–17)	9 (5–14)	0.976
Pain	None—4	None—9	0.100
	Mild—3	Mild—3	>0.999
	Moderate—5	Moderate—0	***0.037***
	Severe—0	Severe—0	>0.999
Clinical range of motion and gait			
Internal rotation (°)	21 (15–27)	43 (31–54)	***0.012***
External rotation (°)	48 (40–55)	42 (33–51)	0.626
Abduction (°)	30 (25–35)	58 (52–64)	**<** ***0.005***
Impingement	9	2	***0.012***
Limp	8	1	***0.009***
Trendelenburg sign	8	1	***0.009***

All continuous data presented as means with 95 % CI in parentheses

All categorical data presented as absolute values

Bolded *p* values represent statistically significant findings (*p* < 0.05)

At the time of surgery, all patients were in the fragmentation phase of the disease on radiographs. Along with femoral head flattening, femoral neck changes were often observed. Mild acetabular dysplasia, compared to the contralateral hip, was evident in 6 of 12 hips based on an LCEA less than 20° (avg. 13°; SD 8.4°). Preoperatively, Shenton’s line was disrupted in 5 of 12 hips and lateral uncovering was noted on plain radiographs based on the extrusion index (avg. 0.75, SD 0.06) and confirmed at the time of intraoperative arthrography.

**Fig. 2:**
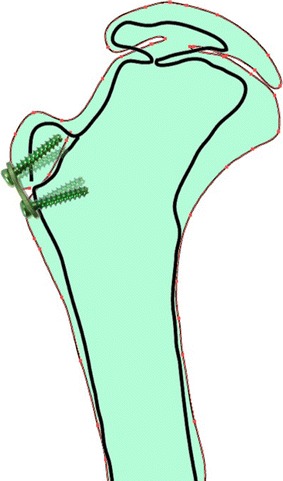
Traced drawing of preoperative and latest follow-up radiographs demonstrating tethering of greater trochanter and relative lack of change to the neck-shaft angle

At latest follow-up, radiographs demonstrated that Shenton’s line was restored in 4 of the 5 hips, and there was no significant worsening in the lateral coverage of the femoral head based upon the measured EI or LCEA. The center head to trochanteric distance (CTD) did not change significantly at latest follow-up and, most notably, we did not see relative overgrowth of the greater trochanter as this would have caused a significant change in the CTD. Neck-shaft angles were not significantly changed from before surgery and, again, we did not see the development of an iatrogenic femoral varus (Fig. 2).

As a group, leg length discrepancy did not change significantly and, at latest follow-up, leg lengths were found to be within 20 mm of the opposite extremity in all hips (mean 9 mm, range 0–20 mm). We did find that leg length discrepancy worsened in four patients, and these patients were treated with contralateral eight-plate distal femoral epiphysiodesis. (Table 2)

Despite its position on the lateral aspect of the greater trochanter, the eight-plate was well tolerated generally. None of the hardware fatigued or broke. Six plates have been removed after an average of 43 months; all other plates were left in situ pending further growth or symptoms. We continue to follow each child bi-annually until maturity to monitor containment, limb lengths and abductor function.

There were no perioperative complications related to wound healing or the Petrie cast. Two patients required a repeat adductor tenotomy at 5 months and 3 years post-index surgery, respectively, for loss of hip abduction. One patient went on to have a greater trochanteric transfer 6 years following the index procedure. Four patients have undergone contralateral epiphysiodesis of the distal femur due to limb length discrepancy. In the remainder, no trochanteric transfers or osteotomies are anticipated.

## Discussion

The femoral neck shares the same intracapsular blood supply as the femoral head. In LCPD, it is common to see radiolucent defects in the femoral neck and, over time, for the neck to become broad and short (coxa brevis). The term “functional coxa vara” is inaccurate and cannot be measured radiographically because the femoral neck-shaft angle does not change appreciably. What is seen is a loss of abductor power as trochanteric growth continues unabated and the lever arm becomes shortened. Instead of an angular deformity, this is a length disturbance occurring proximal to the lesser trochanter. The loss of abductor power is manifest by abductor fatigue pain and a positive Trendelenburg gait. Acetabular dysplasia with or without impingement may ensue [9, 12]. With coxa magna, the diameter of the femoral head increases and moves the center of rotation of the hip distally, relative to the tip of the greater trochanter, thus exacerbating the lever arm compromise of the abductors.

Treatment for LCPD in the fragmentation stage focuses on containment of the femoral head within the acetabulum during the fragmentation stage [16]. Containment may be achieved by non-surgical and surgical methods. The most popular operative methods for containment include osteotomy of the pelvis or femur (or sometimes both) [17], though recently acetabular augmentation (shelf procedure) has enjoyed renewed interest [18, 19]. While coverage, containment, and congruity of the femoro-acetabular joint are of paramount importance, one must be mindful of the role that future femoral neck growth plays with respect to the long-term outcome.

Each of the patients met our selection criteria for surgical containment including progressive limp, increasing pain, failure to improve with observation and symptomatic treatment. The radiographs demonstrated femoral head fragmentation, loss of containment and a break in Shenton’s line. From these combined features, we have assumed the natural history for these children was ominous and, if treated non-operatively, the outcomes would have been poor. Rather than take the popular approach of attempted containment by intertrochanteric osteotomy, we chose another approach.

In addition to the usual goals of containment and range of motion, the holistic approach to managing LCPD should include considerations of abductor strength, acetabular dysplasia and limb length. Pelvic and femoral osteotomies, as well as shelf procedures fail to address these problems and, in fact, may exacerbate them. While the reported results are satisfactory in the short term, few series follow these patients to maturity; revision and secondary surgery for femoro-acetabular impingement, coxa vara or acetabular dysplasia are commonplace.

Proponents of the Salter innominate osteotomy cite improved anterolateral coverage of the femoral head [16, 20]. Detractors have expressed concern about causing increased pressure on the femoral head or eventual femoral-acetabular impingement with or without hinge abduction. Shelf procedures (augmentation arthroplasty) are touted as a less invasive approach [18]. Nevertheless, the abductors have to be stripped in order to properly position and secure the bone graft. Furthermore, the long-term effects of covering over the labrum are unknown, and the risk of creating an anterolateral pincer-type impingement makes this a less attractive option.

Considering that LCPD represents a deformity of length, the iatrogenic varus created by intertrochanteric osteotomy for the sole purpose of achieving containment is concerning. Varus intertrochanteric osteotomy may further aggravate weakening the hip abductors by elevation of the greater trochanter and shortening of the limb. Furthermore, Brown et al. noted, in a finite element analysis, a varus osteotomy increased shear stresses where the lateral epiphyseal artery enters the femoral head and could lead to increased vessel occlusion and further vascular insults to the already tenuous vascularity of the head [1]. Furthermore the older the child, the more likely that iatrogenic varus, will persist and require a secondary valgus intertrochanteric osteotomy to correct it.

**Fig. 3:**
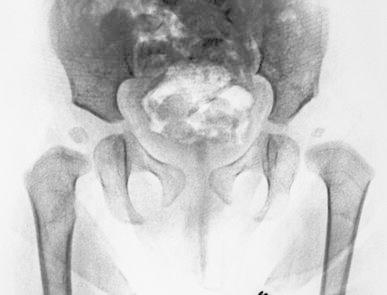
Inverted contrast anteroposterior pelvis radiograph demonstrating the common proximal femoral physis; *O’Brien’s Line*

Recognizing that LCPD represents dysplasia involving the medial 2/3 of the common proximal femoral chondroepiphysis (Fig. 3), we have chosen to tether the greater trochanter physis, at the time of achieving containment, as part of our non-osteotomy management strategy for selected children with progressive symptomatology and related radiographic changes. Tethering of the greater trochanter is convenient to perform at the time of examination under anesthesia, arthrogram, and open adductor and iliopsoas tenotomy. We chose a lateral flexible tether because, when compared to ablative procedures such as a Phemister bone block, drilling or a vertical screw, the femoral neck growth is optimized rather than restricted. This was reflected in the screw divergence noted at follow-up.

The aim of trochanteric tethering (“guided growth” of the proximal femur) is to postpone, or possibly prevent, the need for trochanteric transfer or abduction intertrochanteric osteotomy. By avoiding osteotomy of the pelvis or femur, the abductor length and power are optimized and the relative limb lengths reserved. Undertaking this between the skeletal ages of 5–8, we hope to facilitate growth of the femoral neck portion of the common femoral physis and avert a relative coxa brevis.

In this series, there was a significant improvement in clinical examination findings including hip motion (internal rotation and abduction), decreased impingement, as well as less limping and abductor weakness at latest follow-up. We did not find significant changes in the center edge angle, extrusion index and Shenton’s line, lending evidence to a successful containment. Additionally, there was no significant change in neck-shaft angles or the center trochanteric distance, illustrating a propensity to avoid varus or valgus deformity using this technique as well as a successful tethering of the trochanter, respectively.

We acknowledge that our study has limitations. Lacking objective quantification in testing of abductor strength, we relied upon parental history (limping vs. no limp) and upon the fatigue Trendelenburg test (reliable to experienced clinician) noting that both were improved at follow-up. Secondly, we reviewed a comparatively small number of subjects with relatively short follow-up. Having transferred the greater trochanter in one patient to date, we remain vigilant on behalf of the remainder. However, it appears unlikely that this will become necessary. We intend to continue the follow-up to maturity and are watchful for issues related to congruity, abductor function, acetabular dysplasia and limb lengths. Meanwhile, we have avoided some of the iatrogenic problems that result from more traditional methods of surgical containment. The morbidity of this approach is, by comparison, minimal and the benefits seem to accrue with continued growth.

In conclusion, we have shown that prophylactic guided growth of the greater trochanter at the time of containment procedures minimizes trochanteric overgrowth and improves abductor strength while avoiding iatrogenic deformities of the proximal femur. We believe that adding a trochanteric apophysiodesis to containment-attaining soft tissue release surgery for the hip with LCPD may avoid the deformity of a high-riding greater trochanter and the implications it has on abductor function and impingement.
